# Detection of the minimum concentrations of α-lactose solution using high-power THz-ATR spectroscopy

**DOI:** 10.3389/fbioe.2023.1170218

**Published:** 2023-03-23

**Authors:** Haiqing Wang, Wei Shi, Lei Hou, Chunhui Li, Yusong Zhang, Lei Yang, Juncheng Cao

**Affiliations:** ^1^ Key Laboratory of Ultrafast Photoelectric Technology and Terahertz Science in Shaanxi, Xi’an University of Technology, Xi’an, China; ^2^ School of Physics and Opto-Electronic Technology, Baoji Key Laboratory of Micro-Nano Optoelectronics and Terahertz Technology, Baoji University of Arts and Sciences, Baoji, Shaanxi, China; ^3^ Country the Key Laboratory of Terahertz Solid-State Technology, Shanghai Institute of Microsystem and Information Technology, Chinese Academy of Sciences, Shanghai, China

**Keywords:** high-power THz-ATR spectroscopy, α-lactose solution, signal-to-noise ratio (SNR), LT-GaAs PCA, high-energy LiNbO3

## Abstract

Terahertz (THz) technology has emerged as a promising tool for the qualitative and quantitative identification of markers containing major diseases, enabling early diagnosis and staged treatment of diseases. Nevertheless, the detection of water-containing biological samples is facing significant challenges due to limitations in high-power THz radiation sources and high-sensitivity detection devices. In this paper, we present a designed and constructed set of Terahertz-Attenuated Total Reflection (THz-ATR) spectrometer for high-sensitivity detection of liquid biological samples, which can dynamically maintain the signal-to-noise ratio (SNR) of THz detection signal of liquid biological samples at 40–60 dB. Our high-power THz-ATR spectroscopy can identify and quantitatively detect α-lactose aqueous solution with a minimum concentration of 0.292 mol/L. Moreover, we observed that the rate of change in the absorption peak position varied greatly between high and low concentration samples. Our high-power, high-sensitivity THz-ATR spectroscopy detection provides a rapid, accurate, and low-cost method for detecting disease markers such as blood and urine indicators. Additionally, this approach offers new perspectives for the refinement and in-depth detection of biomedical samples.

## 1 Introduction

Terahertz (THz) spectroscopy has shown great potential in the biomedical field for qualitative and quantitative identification of key substances, such as early detection of cancer and blood indicator detection (Torii et al., 2017; Wang et al., 2020; Shi et al., 2021; Liao et al., 2022). However, the detection of characteristic spectra of target substances is often limited by the insufficient signal-to-noise ratio (SNR) of the detection system, resulting in inaccurate identification of the target substance (Naftaly, 2012; Peng et al., 2020a; Peng et al., 2020b). Factors affecting the SNR of biomedical sample detection include: 1) low SNR of the THz spectroscopy system (Alfihed et al., 2020); 2) strong absorption of THz waves by water, leading to high loss in the transmission of the sample signal (Yang et al., 2016); and 3) interference of non-target materials, leading to difficulties in resolving the target signal (Peng et al., 2018). Therefore, improving the SNR of aqueous samples is fundamental to achieve qualitative and quantitative detection of biomedical samples.

Currently, there are three main technical means to improve the SNR of water-containing samples. The first is system optimization, which involves developing a THz radiation source with high radiated power and good stability. Strong field THz radiation technology includes the use of femtosecond laser and non-linear crystal interaction, high-intensity THz pulse obtained using the tilted pulse-front technique, and the high-power crystal radiation source represented by LiNbO3 crystal, which has been used in experimental detection work (Moriguchi et al., 2018; Yang et al., 2020). Although the interaction of ultrafast and ultra-strong laser and plasma, as well as the new mechanism of strong laser-solid-target interaction, have achieved high radiation power, they have not yet been applied to THz spectroscopy (Yardimci et al., 2017; Liao et al., 2019).

The second approach is the use of THz detection devices to enhance biological THz signals. Existing works focus on methods that utilize evanescent waves or enhance local THz field strength, such as the use of microfluidic chips, metamaterial sensors, waveguides, and THz-ATR (Liu et al., 2019; Hou et al., 2021; Yang et al., 2021; Yu et al., 2022). THz-ATR has several advantages, including high detection sensitivity, low sample requirements, and the ability to achieve qualitative and quantitative detection of samples simultaneously.

The third approach involves algorithm denoising reconstruction of data. For signals containing noise, multiple summations can be used for denoising, and data smoothing methods can be employed, including conventional methods represented by N-point averaging and special methods represented by wavelet transform denoising methods, which may sacrifice some signal information (Qiao et al., 2017; Cui et al., 2019; Takagi et al., 2020).

In this paper, we present a set of THz detection spectrometers for cells and biomolecules that can switch instantly between the low temperature grown GaAs photoconductive antenna (LT-GaAs PCA) radiation source and high-energy LiNbO3 radiation source. ATR-assisted enhancement of sample signals maximizes the detection sensitivity of aqueous samples. With improved system performance, the SNR of different concentration sample signals can be dynamically maintained at 40–60 dB, enabling the qualitative and quantitative detection.

## 2 Sample preparation and experiment

### 2.1 Sample preparation

The α-lactose monohydrate used in this study was procured from Aladdin (Shanghai) Co., Ltd. and was of analytical purity. To ensure accuracy, the sample powder was weighed using an electronic analytical balance with a precision of 0.01 mg. Quantitative addition of water was performed using a pipette gun with a range of 10–100 μl and an accuracy of 1 μl. The α-lactose monohydrate powder to be tested was placed directly into the sample cell on the ATR prism, followed by the quantitative addition of water for the test. As α-lactose monohydrate is soluble in water, the homogeneity of the sample solution was ensured. The concentrations of the prepared samples were 19.467 mol/L, 5.309 mol/L, 1.168 mol/L, 0.664 mol/L, 0.314 mol/L, and 0.292 mol/L, respectively. To prevent water from spreading to the surroundings, a sealing ring was placed on the surface of the ATR prism. During the test, the drip port was sealed with a metal foil to prevent water evaporation, thereby avoiding concentration deviations caused by the test sample.

### 2.2 Design of attenuating total reflection prism

The design of the attenuated total reflection spectroscopic detection system is based on the principle of total reflection of light. When a light wave is incident from a medium with a high refractive index (prism material) to a medium with a lower refractive index (air or aqueous biological samples), total reflection occurs at the interface between the two media if the angle of incidence is greater than the critical angle, also known as the Broster angle. This results in the generation of evanescent waves at the interface between the two media, where the amplitude of the evanescent wave decays exponentially with the vertical direction of the interface. The penetration depth of the incident evanescent wave into the sample can be calculated using the following formula:

$$d_{p}=\frac{\lambda }{2\pi \sqrt{n_{1}^{2}\sin^{2}\theta -n_{2}^{2}}}$$ (1)

where λ represents the wavelength of the incident light wave, n1 and n2 represent the refractive indices of the two media, respectively, and they satisfy n1 > n2. θ is the angle of incidence. The attenuation of the evanescent wave at the interface allows for spectral information to be obtained from a micron or thinner layer from the interface, which can be used for the detection of trace biological samples.

**FIGURE 1:**
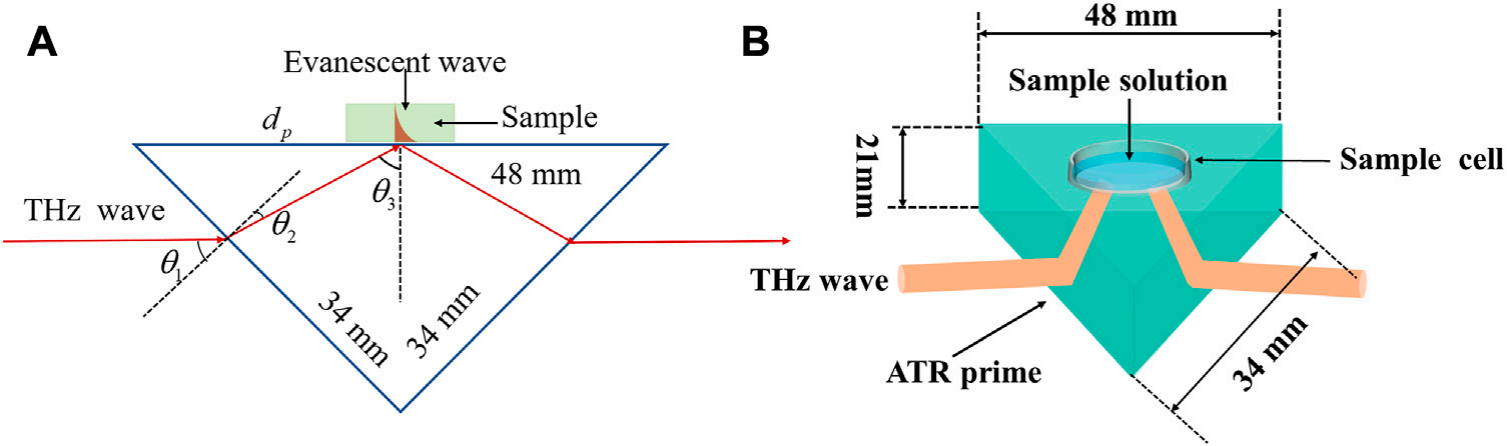
The schematic diagram of **(A)** the design dimensions of the ATR prism and **(B)** THz-ATR spectroscopic detection of aqueous samples.

In this project, molten silicon with a resistance greater than 10,000 Ω/cm is used as the basic material to fabricate the ATR prisms. The ATR prisms are made using cutting, grinding, and polishing processes to form right isosceles triangular prisms. Figures 1A,B show the schematic diagram of the design dimensions of the ATR prism and THz-ATR spectroscopic detection of aqueous samples, respectively. The triangular prisms have side lengths of 48 mm, 34 mm, and 34 mm, respectively, with a thickness of 21 mm. The angle of incidence θ1 is 45°, the angle of refraction θ2 is 11.8°, and the internal total reflection angle θ3 is 56.8°.

### 2.3 THz detection spectrometer for liquid biological samples

We have designed and constructed a THz detection spectrometer for liquid biological samples to meet the high SNR requirements of biomedical sample detection signals. The system consists of two sets of THz radiation sources that can be instantly switched by folding mirrors to meet the detection needs of various samples. Moreover, an ATR setup is employed to enhance the sample signal, ensuring that the SNR of the signal of different concentrations always stays between 40–60 dB to achieve qualitative and quantitative detection of aqueous samples.

**FIGURE 2:**
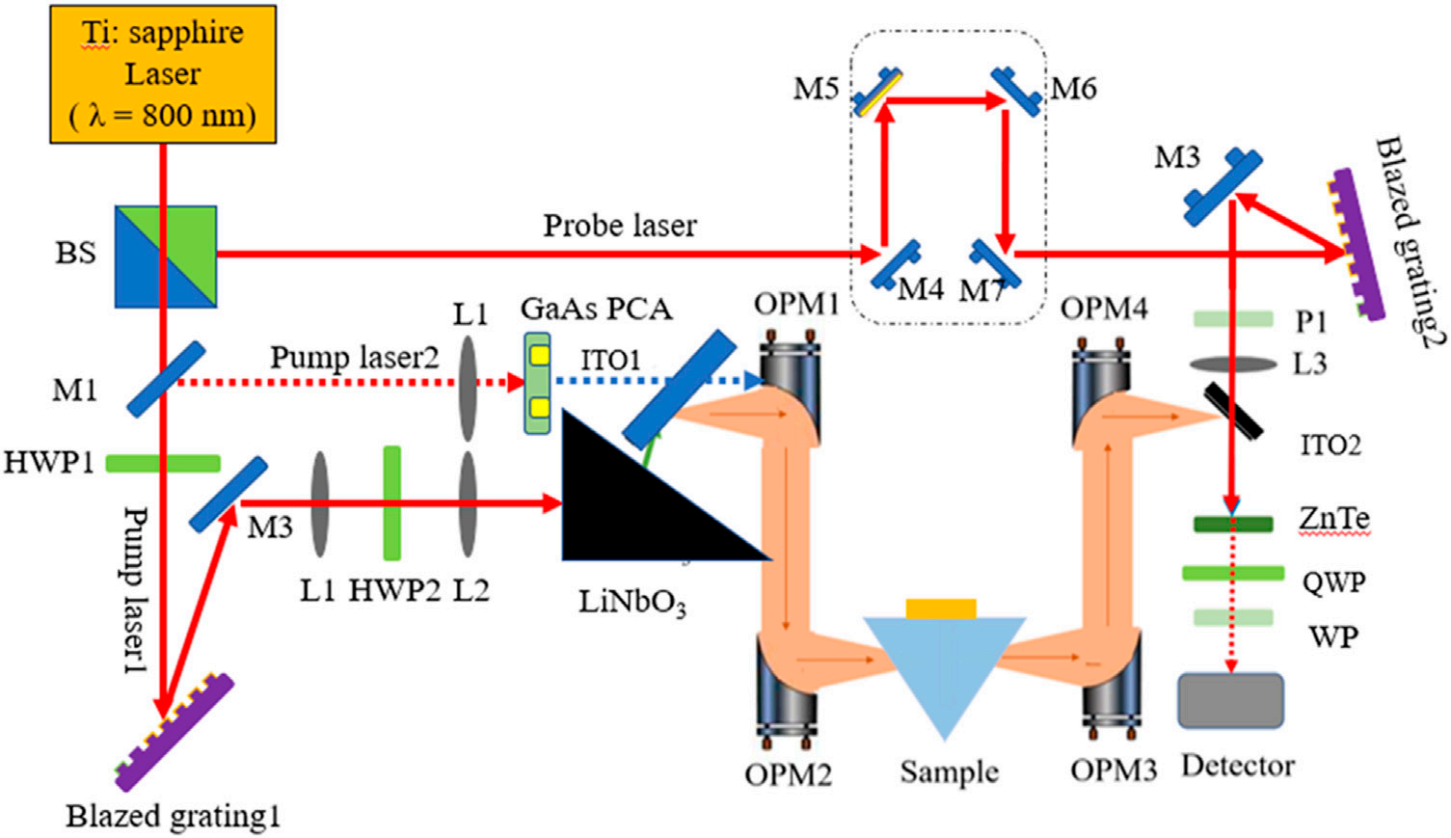
The schematic diagram of the design optical path of THz-ATR spectrometer for liquid biological samples.

The schematic diagram of the designed optical path of the THz-ATR spectrometer for liquid biological samples is presented in Figure 2. The system mainly comprises a MaiTai femtosecond laser, THz radiation sources (LT-GaAs PCA and LiNbO3 crystal), ATR prism, and THz detector. The MaiTai XF-1 titanium sapphire femtosecond laser oscillator from Spectra-Physics is used as the seed light source, with an average output power of approximately 4.5 W. The femtosecond laser is split into pump light 1 and probe light by a beam splitter, with corresponding pulse energies of 3 W and 100 mW, respectively. Pump 1 is attenuated and then passed through mirror M1 (foldback mirror) to generate pump light 2 with a pulse energy of about 300 mW.

First, the laser polarization direction of pump light 1 is adjusted to horizontal polarization through the half-wave plate HWP1 to maximize the first-stage diffraction efficiency of the blazed grating. Then, the first-stage diffraction of the shining grating creates an inclined wavefront, which is vertically polarized by adjusting the direction of laser polarization through the half-plate HWP2. Finally, pump light 1 is incident at a suitable wavefront inclination angle to a LiNbO3 crystal with a cutting angle of 63°, matching the wave velocity of the THz wave and the femtosecond laser in the exit direction of the THz wave, thus exciting a high-power THz wave. Pump light 2 is focused through the lens and incident on the LT-GaAs PCA to generate broad-spectrum THz waves. The THz pulses exiting from the radiation sources are collimated by the first off-axis parabolic mirror (OPM1) and then converged by the off-axis parabolic mirror (OPM2). The ATR sample cell is placed at the focal point of the second off-axis parabolic mirror. The reflected THz waves carrying the sample information are collected by OPM3 and OPM4 and focused on ZnTe. Finally, data acquisition is carried out by lock-in amplifier and computer. The entire system is placed in a closed box and filled with dry air to ensure that the relative humidity of the working environment is less than 3%, and the working environment temperature is maintained at 24°C.

In conclusion, we have successfully designed and constructed a THz detection spectrometer for liquid biological samples, capable of achieving qualitative and quantitative detection of aqueous samples. The system’s two sets of THz radiation sources and ATR setup ensure that the SNR of the signal of different concentrations is always maintained between 40–60 dB, meeting the high SNR requirements of biomedical sample detection signals. The system’s construction and design, as well as its performance, have been described in detail in this paper.

## 3 Results and discussion

### 3.1 THz-ATR spectroscopy detection

**FIGURE 3:**
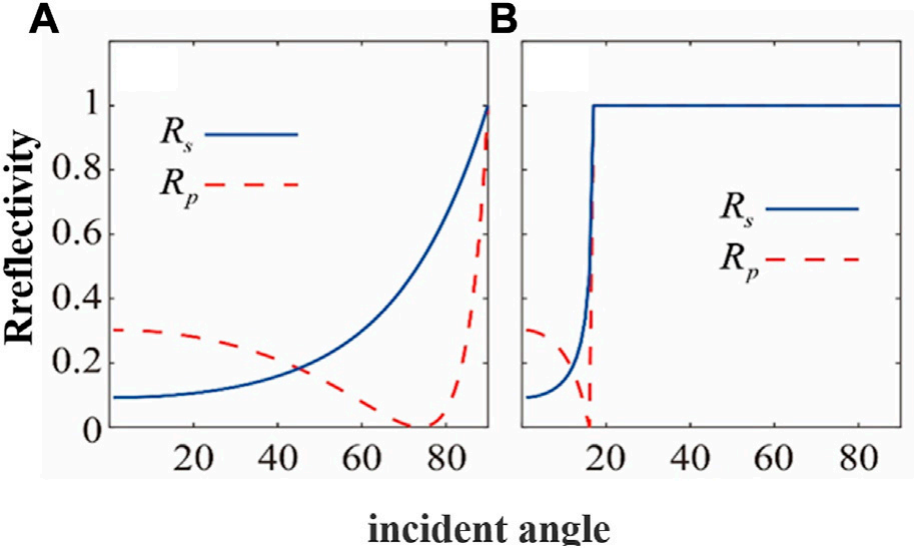
The reflectivity of S-light and P-light **(A)** incident from air to high-resistivity silicon and **(B)** incident from high-resistivity silicon to air.

In the THz band, high-resistivity silicon exhibits desirable characteristics such as a high refractive index (∼3.45), small propagation loss, and minimal dispersion, making it a widely-used material in device processing for THz photonics research. The reflectivity of S and P light when air is incident on high-resistivity silicon and *vice versa* are shown in Figures 3A, B, respectively. This indicates that only the S-light incident on ATR prism can achieve total reflection of THz light and generate the evanescent wave necessary for sample detection. Furthermore, an incidence angle of 45° for S-light ensures minimal loss of high-resistance silicon to THz waves, as well as minimal outgoing sample THz signal loss. Additionally, the total internal reflection angle of 56.8° ensures almost all focused incident light will produce total reflection.

**FIGURE 4:**
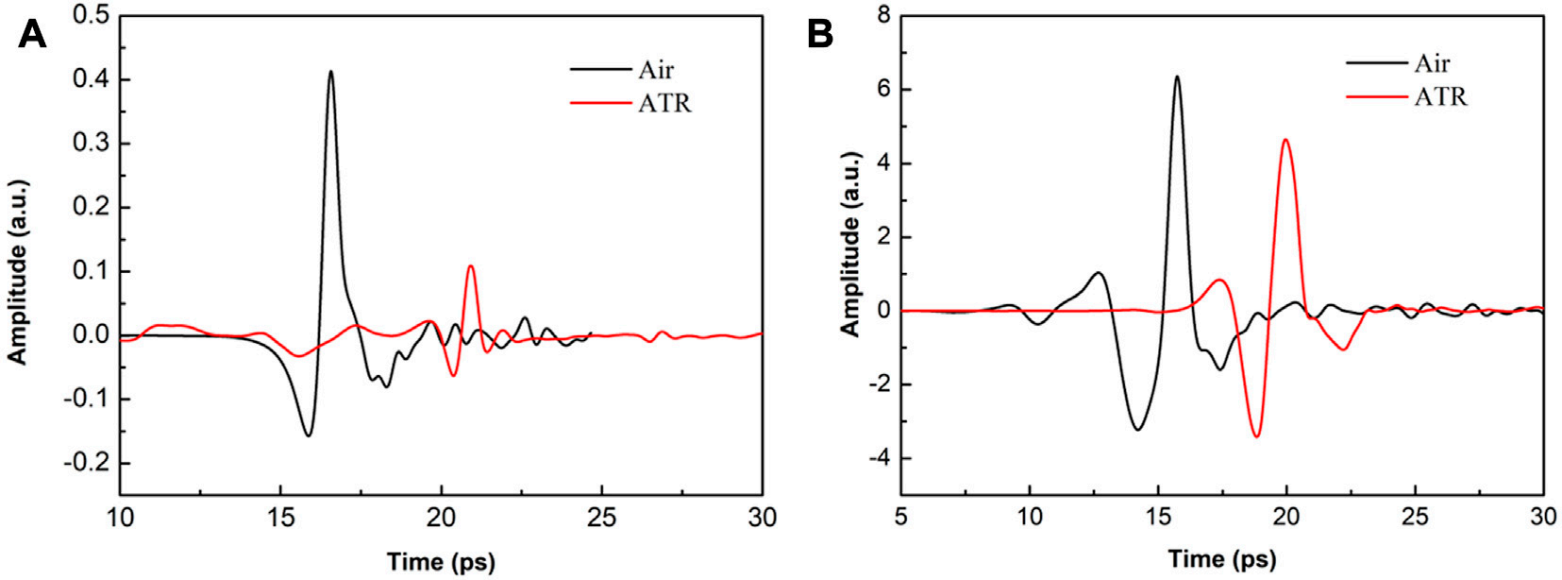
THz time-domain spectral comparison diagram of system and no-load ATR prime, when the radiation source is **(A)** LT- GaAs PCA and **(B)** high-energy LiNbO3, respectively.

Taking these factors into account, experiments have shown that the complete time-domain spectrum of THz-ATR can be detected regardless of whether an LT-GaAs PCA or high-energy LiNbO3 radiation source is used, as shown in Figures 4A, B. Notably, the ordinates of both figures have the same magnification for detection amplitude. As the ATR prism has a path delay of 12 mm, the abscissa of the ATR signal is moved forward for ease of comparison. Comparing Figures 4A, B, it can be observed that the peak-to-peak THz signal of high-energy systems and ATR prisms is 17.42 times and 41.5 times higher than that of low-energy radiation THz, respectively. Furthermore, the effective utilization rate of the THz-ATR signal of the high-energy LiNbO3 source is about 80%, which is much higher than the effective utilization rate of the PCA source of 30%.

Two factors contribute to the utilization difference: 1) for LiNbO3 crystals characterized by low-frequency and high-power, the low-frequency signal concentrated in the range of 0.1-1 THz is very strong, and a small amount of low-frequency signal loss will not cause an insufficient SNR due to the reflection of the ATR prism; 2) for LT-GaAs PCA with low-power and wide-spectrum radiation characteristics, in the range of 0.1-3 THz, strong low-frequency signals and weak high-frequency signals are present. The ATR reflection loss will cause the loss of some high-frequency signals, resulting in decreased SNR. However, due to the different requirements of THz radiation power and spectrum for test samples, the choice of detection method should be made according to the specific advantages of each.

### 3.2 Detection of different concentrations of α-lactose solution using traditional THz-ATR spectroscopy

Traditional THz-ATR systems have shown potential for quantitative detection of high concentrations of α-lactose samples. However, these systems cannot distinguish between different types of α-lactose molecules due to the low SNR. In this study, we investigated the effect of water content on the THz spectra of α-lactose solutions using THz-ATR spectroscopy.

**FIGURE 5:**
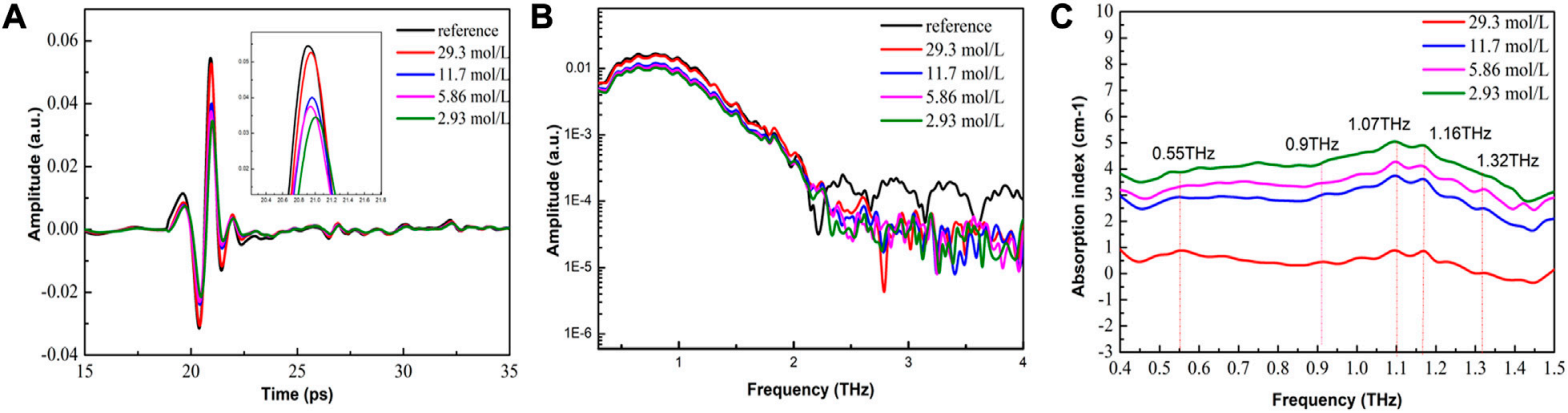
THz-ATR spectroscopy of reference (no-load ATR sample cell) and α-lactose solution with different concentrations. **(A)** Time-domain spectra (see enlarged image for details); **(B)** The corresponding frequency-domain spectra; and **(C)** Characteristic absorption spectra.

As shown in Figure 5A, the THz signal of α-lactose samples exhibits a clear time delay effect compared to the reference signal of the no-load ATR sample cell. This effect is caused by the refraction of THz waves through the sample, and becomes more pronounced as the water content in the sample increases. Moreover, as the water content increases, the signal amplitude of the sample decreases correspondingly. The frequency-domain spectra of Figure 5B show that the signal amplitude of the sample also decreases significantly in the range of 0.1-2 THz with increasing water content. By combining these observations with the characteristic absorption spectra of Figure 5C, we can distinguish between different concentrations of α-lactose solutions, but cannot distinguish the absorption peak of α-lactose.

We attribute this phenomenon to three factors. Firstly, the penetration depth of the evanescent wave incident into the sample is only tens of microns, which results in ATR spectra that provide only micron-level spectral information. Secondly, the amplitude of the sample absorption peak is determined by the superposition effect of the sample molecules absorbing THz waves, which can result in the spectral fingerprinting features of THz being weakly masked by noise due to the shallow THz penetration depth. Thirdly, ATR prisms are not specific for sample signal enhancement, which can result in the water signal being enhanced and the α-lactose sample signal being masked with a low SNR.

Notably, the strong absorption of water is extremely sensitive in the detection spectrum, which enables quantitative detection of high concentrations of α-lactose solutions based on different absorption baselines of water content. Overall, our results highlight the potential of THz-ATR spectroscopy for studying the effects of water content on the THz spectra of α-lactose solutions.

### 3.3 Detection of different concentrations of α-lactose solution using high-power THz-ATR spectroscopy

The detection of low-concentration aqueous biological samples is a significant challenge due to the strong absorption of water, which masks the absorption peak of the sample, making it difficult to identify and quantify the sample. However, the use of high-intensity THz radiation power has been shown to improve the SNR of the reflected signal of aqueous samples, resulting in improved detection accuracy. Furthermore, THz waves generate evanescent waves at the ATR-Sample interface, which extend into and interact with the sample, achieving highly sensitive detection of micro samples. THz-ATR spectroscopy shows higher sensitivity than ordinary THz spectroscopy, making it ideal for detecting low-concentration aqueous samples.

**FIGURE 6:**
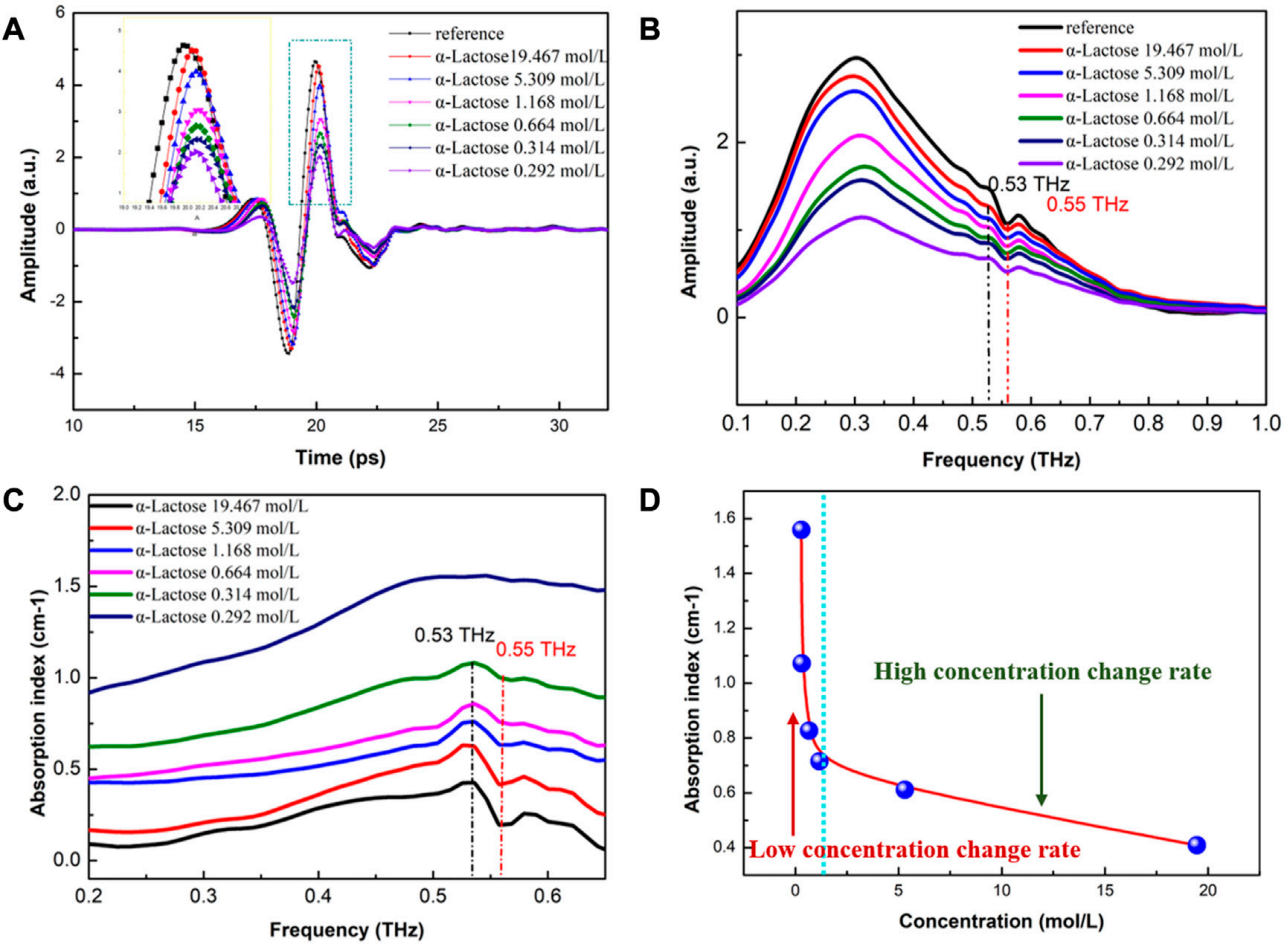
High-power THz-ATR spectra of α-lactose samples with different concentrations **(A)** Time-domain spectra (see enlarged image for details); **(B)** The corresponding frequency-domain spectra; and **(C)** Characteristic absorption spectra; and **(D)** The change rate of absorption peak position with different concentrations of α-lactose solution.

In this study, high-power THz-ATR time-domain spectra were obtained for α-lactose samples with different concentrations, with the minimum concentration reaching 0.292 mol/L. From Figures 6A,B, it can be found that the decrease in concentration resulted in a delay effect and amplitude reduction of the sample signal, indicating that the detection spectrum carried sample information. The SNR of the sample signal was maintained at 40 dB–60 dB for different concentrations, providing the possibility for the qualitative and quantitative detection of low-concentration aqueous samples. The frequency-domain spectra were smooth, with minimal noise impact on the sample signal. This is because the power of the THz wave passing through the sample is relatively high, and ATR enhances the sample signal, greatly improving the sensitivity of sample detection. A significant drop point was observed in the spectra of the reference and samples at the frequency position of 0.55 THz, while only the sample spectra had a particularly small drop point at 0.53 THz.

The characteristic absorption spectra of α-lactose aqueous solution were analyzed, enabling the identification and quantitative detection of the solution with a minimum concentration of 0.292 mol/L, which is presented in Figure 6C. A normal absorption peak was observed at 0.53 THz, while a concave peak was observed at 0.55 THz, with the concave peak decreasing with the decrease in concentration. The absorption baseline of the entire absorption spectra became higher overall as the concentration decreased. The peak of 0.53 THz was identified as the absorption peak of α-lactose molecules, while the peak at 0.55 THz may have been caused by water vapor. However, the test environment was dry air with humidity lower than 3%, and previous high-power THz detections based on the sample cell had no interference of water vapor (Wang et al., 2023). Therefore, it is speculated that the observed phenomenon was caused by the enhancement effect of ATR on the sample signal, and ATR is more sensitive than the sample cell. As shown in Figure 6D, the change rate of the absorption peak position with different concentrations of α-lactose solution was analyzed, revealing a linear inverse relationship between the absorption peak position and the decrease in sample concentration at high concentrations. However, at low concentrations, the absorption peak position showed a non-linear inverse relationship with the decrease in sample concentration. The rate of change in the absorption peak position varied greatly between high and low concentration samples, possibly due to the increasing contribution of water to the absorption rate and the gradual weakening of the contribution of samples.

In conclusion, the use of high-power THz-ATR spectroscopy can improve the detection sensitivity and SNR of aqueous samples, making it an ideal tool for the qualitative and quantitative detection of low-concentration aqueous samples. The observed characteristics in the spectra provide insights into the molecular interactions of aqueous biological samples, facilitating further studies in the field of biomedical research.

## 4 Conclusion

In this paper, we present the design and construction of a set of THz-ATR spectrometers for highly sensitive detection of liquid biological samples. The system is capable of switching between two types of THz radiation sources, namely, traditional LT-GaAs PCA and high-energy LiNbO3, to meet the detection requirements of different samples. Our experimental results demonstrate that traditional THz-ATR spectroscopy can be employed for the quantitative detection of high concentrations of α-lactose samples, while high-power THz-ATR spectroscopy is effective for the qualitative identification and quantitative detection of α-lactose aqueous solutions with a minimum concentration of 0.292 mol/L.

Moreover, we observe that the absorption peak position changes linearly with the decrease of sample concentration at high concentrations; at low concentrations, the absorption peak position showed a non-linear inverse relationship with the decrease in sample concentration. Furthermore, the change rate of absorption peak position varies significantly between high and low concentration samples. Through the use of high-power THz-ATR technology, we have achieved qualitative identification and quantitative detection of low-concentration α-lactose solutions, which provides a novel approach for THz spectroscopic detection of biomedical samples. In the future, it is anticipated that the qualitative and quantitative identification of low-power and broad-spectrum THz-ATR can be accomplished through effective data denoising technology. Overall, our work contributes to the advancement of THz-ATR spectroscopy and its potential applications in the field of biomedical research.

## Data Availability

The raw data supporting the conclusions of this article will be made available by the authors, without undue reservation.
